# Viral Vectors for the *in Vivo* Delivery of CRISPR Components: Advances and Challenges

**DOI:** 10.3389/fbioe.2022.895713

**Published:** 2022-05-12

**Authors:** Misganaw Asmamaw Mengstie

**Affiliations:** Department of Biochemistry, College of Medicine and Health Sciences, Debre Tabor University, Debre Tabor, Ethiopia

**Keywords:** viral vectors, delivery, CRISPR, Cas-9, genome editing, review

## Abstract

The Clustered Regularly Interspaced Short Palindromic Repeat (CRISPR) and its accompanying protein (Cas9) are now the most effective, efficient, and precise genome editing techniques. Two essential components of the CRISPR/Cas9 system are guide RNA (gRNA) and CRISPR-associated (Cas9) proteins. Choosing and implementing safe and effective delivery systems in the therapeutic application of CRISPR/Cas9 has proven to be a significant problem. For *in vivo* CRISPR/Cas9 delivery, viral vectors are the natural specialists. Due to their higher delivery effectiveness than other delivery methods, vectors such as adenoviral vectors (AdVs), adeno-associated viruses (AAVs), and lentivirus vectors (LVs) are now commonly employed as delivery methods. This review thoroughly examined recent achievements in using a variety of viral vectors as a means of CRISPR/Cas9 delivery, as well as the benefits and limitations of each viral vector. Future thoughts for overcoming the current restrictions and adapting the technology are also discussed.

## Introduction

Genome editing refers to a set of technologies that allow scientists to alter the DNA of an organism (Khalil, 2020). Genome editing technology advancements and promising applications have had a significant impact on basic science and clinical research (Li et al., 2020a). CRISPR (clustered regularly interspaced short palindromic repeats) CRISPR-associated protein (Cas9) was derived from a naturally occurring genome editing system in bacteria. The two components in CRISPR are guide RNA (which is used to locate/bind the target DNA to be edited) and Cas9 (a protein that essentially cuts the DNA at the location identified by guide RNA) (Desai et al., 2021). CRISPR RNA (crRNA) and trans-activating CRISPR RNA (tracrRNA) are the two parts of guide RNA. The crRNA is an 18–20 base pair RNA complementary to the target DNA, whereas tracrRNA is a lengthy stretch of loops that serves as a Cas nuclease binding scaffold (Marx, 2020). It can be synthesized by combining crRNA and tracrRNA to generate a single guide RNA (sgRNA) to target the gene sequence in the gene-editing tool (Allen et al., 2021). Even though CRISPR/Cas9 has numerous roles ranging from basic molecular research to clinical applications, one of the current challenges of this technology is the lack of safe and effective delivery methods (Asmamaw and Zawdie, 2021). In CRISPR/Cas9 delivery methods, both the vehicle (the method of delivery into cells) and the cargo (Cas9 and sgRNA) are involved. Viral and non-viral modalities for delivering CRISPR components to the target cell/tissue have been developed by researchers (Lino et al., 2018). Due to their high cellular uptake and editing efficiency, viral vectors are currently regarded as the natural experts of *in vivo* CRISPR delivery relative to the other methods (Wilbie et al., 2019). However, there are still some common hurdles associated with the viral delivery of CRISPR/Cas9 components, like insertional mutagenesis, immunogenicity, and off-target effects (Kotagama et al., 2019). This review looked at the most widely studied viral vectors such as adeno-associated viral vectors, lentivirus vectors, and full-sized adenoviral vectors, and the recent progress in viral delivery mechanisms, as well as the benefits and drawbacks of each method. Recent potential advancements in the clinical application of CRISPR/Cas9 were also mentioned.

### Adeno Associated Viral Vectors

Adeno-associated Viruses (AAVs), which are small, non-enveloped, non-pathogenic single-stranded DNA viruses from the Parvoviridae family, are gaining popularity due to their potential use as vectors (Naso et al., 2017). Despite the high prevalence of these viruses in the human population (approximately 80% of humans are AAVs seropositive), it has not been linked to any human disease. Due to its low immunogenicity and cytotoxicity, and limited integration into the host cell, it is currently the leading *in vivo* delivery system of CRISPR components compared to other viral methods (Verdera et al., 2020; Asmamaw and Zawdie, 2021). Another key advantage of AAV vectors over other viral vectors is that there are several known serotypes of AAV for different tissue tropisms, such as epithelial lung cells, heart cells, neurons, and skeletal muscle cells, which makes tissue-specific delivery much easier (Wilson and Gilbert, 2018).

Although the virus can integrate into the host genome to some extent, limiting its application, a recent advancement called recombinant AAV (rAAV) vector ablates the problem (Bijlani et al., 2022). The AAV has replication (Rep) genes encoded to produce Rep proteins such as Rep78, Rep68, Rep52, and Rep40. These proteins are required for viral DNA replication, modulating viral gene expression, and site-specific integration into the host genome (Vogel, 2013). Specifically, the AAV Rep78/68 proteins have been shown to play a significant role in site-specific integration (Musayev et al., 2015). However, rAAV is produced by removing these Rep genes from the viral genome, thus integration of rAAV in the absence of Rep proteins is inefficient (Dobrowsky et al., 2021). In a preclinical trial, for example, *in vivo* delivery of CRISPR/Cas9 via recombinant AAV vectors effectively alleviates atherosclerosis in a Low-Density Lipoprotein Receptor (LDLR) mutant mouse (Zhao et al., 2020). To date, AAV vectors have been used in hundreds of clinical trials and have been approved by the Food and Drug Administration (FDA) in several instances (Mendell et al., 2021). For instance, AAV vector-based delivery of CRISPR/Cas9 has been used successfully to treat a wide range of disease models such as neurodegenerative diseases (Fuentes and Schaffer, 2018), muscular dystrophy (Long et al., 2016; Nelson et al., 2016), metabolic liver diseases (Yin et al., 2020), and sickle cell diseases (Park and Bao, 2021).

Despite this incredible record of accomplishment, the limited virus cloning capacity (due to the limited size of the virus) and the large size of the commonly used *Streptococcus pyogenes* Cas9 (Sp Cas9) protein remain significant barriers to the use of AAV vectors. Therefore, AAV can only carry a smaller genetic cargo of less than 5 kb genetic material due to its small physical size and genome length (Le Rhun et al., 2019). Several attempts have been developed by scientists to address the issue of the AAV cargo limit. The first method is to use AAVs to deliver only the sgRNA into cells that have already been altered to express Cas9 protein. The second method is to infect the sgRNA and Cas9 into separate AAVs with distinct tags, co-transfect them into cells, and then screen the cells for successful co-transfection. However, this dual AAVs delivery method requires a high viral dose that imposes safety concerns and can reduce the therapeutic editing potential (Hayashi et al., 2020; Fang et al., 2021). In addition, the advancement of AAV-mediated base and prime editing systems have been developed to overcome the limitations of traditional Cas9 nuclease. They use catalytically inactivated or dead Cas9 endonuclease (Kantor et al., 2020). Base and prime editors are attractive agents for *in vivo* therapeutic genome editing because they do not generate double-stranded DNA breaks (DSBs), do not require a DNA donor template, and are more efficient in editing non-dividing cells (Anzalone et al., 2020). For instance, researchers recently demonstrated that *in vivo* delivery of the cytidine base editor with Cas9 via dual AAVs effectively treats a mouse model of amyotrophic lateral sclerosis (ALS), one of the neurodegenerative disorders (Lim et al., 2020). Prime editing also further expands the scope of base editing and induces a much lower off-target effect than Cas9 endonuclease (Anzalone et al., 2019). *In vivo* AAVs-mediated delivery of base editing agents was also found to be effective in treating metabolic liver disease (Villiger et al., 2018) and pathogenic mutation of a mouse model (Liu et al., 2021). However, AAV-base and prime-editing technologies for *in vivo* delivery faces the same set of challenges of AAV mediated Cas9 delivery approaches in general, such as vector-induced immunogenicity, therapeutic potency, persistence, and off target effects (Kantor et al., 2020).

The third method to bypass the limitations in cargo size is using smaller Cas9 versions and an alternative class of effector proteins rather than the more commonly used Sp Cas9 (Uddin et al., 2020). Smaller Cas9 versions discovered in recent years such as *Streptococcus aureus* (SaCas9) and *Streptococcus thermophiles* (St1 Cas9) are important to allow the packaging of sgRNA and Cas9 in the same AAVs (Wang et al., 2020). Cpf1, a class II CRISPR effector, has also recently emerged as an alternative for Cas9 due to its unique properties, including the capacity to target T-rich motifs, the lack of a trans-activating crRNA, and the ability to process both RNA and DNA (Safari et al., 2019). The size of Cpf1 endonuclease is also small compared to Cas9 and is now the most versatile and effective genome editing tool, particularly in plant genome editing (Kim et al., 2017; Alok et al., 2020). Moreover, alternative Cas effector proteins such as Cas13, other than Cas9 are currently used whose delivery of viral vector is relevant. Cas13 nucleases are a type of RNA targeting CRISPR effector protein that can silence target gene expression in mammalian cells (Gupta et al., 2022).

The other method to expand the carrying capacity of AAVs is intein-mediated trans-splicing (Tornabene et al., 2019). Split inteins are a pair of naturally occurring proteins that mediate protein trans-splicing in the same way that an intron in pre-mRNA splicing (Wang et al., 2022). The split AAVs break a large transgene into two pieces and package each piece into an individual AAV. When both AAVs are used to transduce a cell, the full-length gene and/or protein are reconstituted (Chamberlain et al., 2016). Intein-mediated trans-splicing, in particular, has shown a promise in the delivery of base and prime editors (Villiger et al., 2018; Lim and Kim, 2022). Furthermore, several genetically engineered AAV variants have also recently been developed to improve the efficiency and specificity for *in vivo* delivery, despite the need for future improvements in engineered AAV performance (Lau and Suh, 2017).

However, considering that much of the population has been exposed to AAV vectors, researchers have raised another concern about their use: pre-existing immunity against the bacterial Cas9 protein (Ibraheim et al., 2021). Since the CRISPR components are derived from bacteria, the immune system of the host can react to them. As a result, one of the most challenging aspects of the clinical trial is Cas9 protein-specific immunogenicity. However, all viral vectors, not just AAV vectors, could have Cas9-specific immunity (Mehta and Merkel, 2020; Hakim et al., 2021). Immunogenicity against AAV capsid is another immunity-related challenge for this vector system. Because AAV is non-enveloped and has a protein shell, it is very easy for the host immune cells to produce antibodies against it (Verdera et al., 2020). Recently, pre-existing immunity against wild-type AAV has also been observed in healthy donors. This could have a number of negative consequences, including blocking delivery, triggering acute inflammation, or initiating immune system-induced destruction of the edited cells (Li et al., 2020b). Currently, the AAV capsid protein coating can be optimized/engineered through the alteration of antigen site or chimeric AAV capsid to reduce binding affinity to AAV antibodies, thus evading the host immune response. AAV-mediated CRISPR delivery *in vivo* benefits from the flexibility and diversity/serotypes of AAV capsid structure (Ronzitti et al., 2020).

### Lentivirus Vectors

Lentivirus is a retrovirus genus with a single-stranded genome and an enveloped RNA virus. Among the many varieties, the human immune deficiency virus (HIV) is the most commonly utilized and has become the standard lentivirus vector for delivering genetic material to target cells (Milone and O’Doherty, 2018). LVs are another type of viral vector that is being used to deliver CRISPR components. It has a higher cloning capacity than the AAV vector. It can package two copies of an RNA genome (about 10 kilobases), making it a suitable platform for delivering the most common CRISPR/Cas protein (Cas9) and sgRNA cassette with a single viral transfection event (Yip, 2020). LVs vector can be used for both dividing and non-dividing cells. They have also low intrinsic immunogenicity and are inexpensive to scale up their production (Dong and Kantor, 2021). It offers several notable benefits for stem cells in particular. First, it can deliver strong, stable, and permanent exogenous gene expression for random genome integration, which is critical for CRISPR imaging and screening (Henkel et al., 2020). Second, LVs CRISPR-mediated genetic screening often uses a very small ratio of viruses to cells or multiplicity of infection (MOI). The LVs sequences are transcribed to RNA after integration. As a result, stem cells produce hundreds of sgRNAs with less cellular toxicity (Kuhn et al., 2021). Third, lentivirus can be directly added for co-culturing with cells, obviating the damage from cell pretreatment (Zhang et al., 2017). The most difficult challenge associated with the LVs vector system is random integration into the host cell genome using their integrase enzyme. This integration can increase the expression level of proto-oncogenes, leading to cellular transformation (Behr et al., 2021). Viral integration may be advantageous in applications requiring long-term transgenic expression, such as when creating model organisms. However, for therapeutic purposes, LVs pose a safety risk due to insertional mutagenesis and persistent expression of site-specific nucleases, which can result in off-target mutation (Bushman, 2020).

To reduce the risk of integration, researchers have recently designed non-integrating lentivirus vectors (NILVs) for the delivery of CRISPR components by mutating the viral integrase gene or by modifying the attachment sequence of long terminal repeats (LTRs) (Gurumoorthy et al., 2022). They are the most promising delivery tool candidates since they have a high transfer efficiency, superior packaging capacity (compared to other vectors), are expressed transiently and demonstrate very weak integration capability, and do not produce insertional mutagenesis (Ortinski et al., 2017). For instance, delivery of Cas9 protein through NILVs vectors resulted in efficient DNA breakage and genome correction in sickle cell disease (SCD) (Uchida et al., 2021). In addition to their relevance in infectious and genetic diseases, NILVs vectors are being used in many clinical applications, including vaccination, cell type differentiation, and site-directed integration (Rilo-Alvarez et al., 2021; Gurumoorthy et al., 2022). Hence, the transient expression nature of NILVs in dividing cells, combined with their low immunogenicity makes them the most promising vector candidates to be used in clinical applications (Luis, 2020).

### Adenoviral Vectors

Adenoviruses are non-enveloped double-stranded DNA viruses with genomes averaging 36 kb in size and can package around 8 kb of foreign DNA (Ismail et al., 2018). AdVs have been utilized as a vector from the beginning of gene therapy and are one of the best well-studied viruses both biologically and clinically (Gallardo et al., 2021). AdVs vectors have no endogenous machinery and do not integrate into the host genome at high frequency, ablating the risk of off-target effect and insertional mutagenesis seen with other vectors (Li and Lieber, 2019). The AdV can also be used for a targeted knock-in approach. In a pre-clinical study, for instance, AdVs mediated delivery of CRISPR/Cas9 achieved a successful *in vivo* gene knock-in human alpha-1-antitrypsin (Stephens et al., 2018). The AdVs vector-mediated delivery of the CRISPR/Cpf1 system has also been shown to be a useful tool for genome manipulation in human hepatocytes. However, adaptive immune responses against the AdVs and Cas9 were observed (Tsukamoto et al., 2018). AdVs vectors are also not limited in size and can carry much larger amounts of CRISPR cargo. First-generation AdVs vectors with E1 and E3 gene deletions have a packaging capacity of about 8.5 kb, which is nearly double the capacity of AAV vectors (Lee et al., 2017). Recently, researchers have also designed a third-generation high capacity adenoviral (HC-AdVs) vector (also called gutless) through the removal of all viral coding genes to deliver all customized CRISPR components by a single viral vector (Tasca et al., 2020). This method has relevant features to expand the scope of CRISPR/Cas9 delivery such as, extending the cloning capacity of the virus up to 36 kb, having remarkable efficiency of *in vivo* transduction, and production of high titers (Ricobaraza et al., 2020). Hexon, penton, and fiber, the three proteins encapsulating the virus genome, have all been shown to be amenable to genetic manipulation to change the virus features (Beatty and Curiel, 2012). This property, together with clinical trial safety records, makes the AdVs an appealing *in vivo* delivery system for CRISPR components (Boucher et al., 2020). In general, AdVs vectors have a significant cost and scalability advantage over other vectors. Even the more complex gutless AdVs can be produced at a lower cost scale than recombinant AAVs (Srivastava et al., 2021). This advantage is further verified by the fact that AdVs are the leading candidate for the coronavirus disease 2019 (COVID-19) vaccination. This means that the AdVs vaccine can be scaled up to a global scale to prove its utility (Jacob-dolan and Barouch, 2022).

Since this virus is commonly encountered in daily life, the main issue with using AdVs vectors to deliver CRISPR components is that they cause an undesirable immune response in host cells, resulting in tissue inflammation and subsequent removal of the AdVs vector. Furthermore, approximately 90% of human populations have pre-existing antibodies (Lee et al., 2017). Immunity to vectors in CRISPR/Cas9 viral delivery is a significant challenge, lowering the effective viral concentration and necessitating higher vector doses to achieve significant genomic repairs. The immunological response is amplified even more as a result of the higher doses (Chen et al., 2020). Hence, detecting and lowering immunogenicity is one of the most difficult jobs in this viral delivery of CRISPR/Cas9 components. Various strategies, such as AdVs engineering through copolymer encapsulation and the use of non-human AdVs vectors, are now being used to limit the influence of cross-reactive immunity (Lopez-Gordo et al., 2014; Meliani et al., 2018). The production of AdVs is also laborious, which limits the applicability and efficiency of the method for CRISPR delivery.

**TABLE 1:** Summary of the benefits, drawbacks, and current advancements in viral vectors delivery of CRISPR.

Viral vector	Advantage	Disadvantage	Current advances to mitigate the drawbacks
AAVs	Less integration to host genome, variety of serotypes for different cell tropisms	Limited viral packaging capacity, viral capsid immunogenicity	Dual AAVs delivery method (delivering sgRNA and Cas9 separately), smaller Cas9 and alternative Cas effectors (Cas13, Cpf1, prime and base editors), engineering the AAV capsid (altering the antigen site or chimeric AAV capsid)
LVs	High cloning capacity, used for both dividing and non-dividing cells. less immunogenic, and inexpensive production	Random integration to host genome and insertional mutagenesis	Newly engineered non-integrating lentivirus vectors (NILVs)
AdVs	Low integration to host genome, high packaging capacity	High viral-specific immunogenicity	Engineering AdVs through copolymer encapsulation and the use of non-human AdVs vectors

## Conclusion

The most common *in vivo* delivery methods for CRISPR components are viral vectors such as AAVs, LVs, and AdVs. There is no single best viral vector for delivering CRISPR components since each viral vector has advantages and drawbacks in comparison to others. Despite the fact that viral vectors have a high *in vivo* transfection efficiency and new strategies continue to progress, there are still some concerns about their clinical application, such as immunogenicity, integration, and off-target effects.
